# Simultaneous Determination of Volatile Constituents from *Acorus tatarinowii* Schott in Rat Plasma by Gas Chromatography-Mass Spectrometry with Selective Ion Monitoring and Application in Pharmacokinetic Study

**DOI:** 10.1155/2013/949830

**Published:** 2013-11-17

**Authors:** Xue Meng, Xinfeng Zhao, Shixiang Wang, Pu Jia, Yajun Bai, Sha Liao, Xiaohui Zheng

**Affiliations:** ^1^College of Life Sciences, Northwest University, P.O. Box 195, No. 229 Taibai North Road, Xi'an 710069, China; ^2^Key Lab for New Drugs Research of TCM in Shenzhen, Shenzhen 518057, China

## Abstract

A sensitive and specific gas chromatographic-mass spectrometry with selected ion monitoring (GC-MS/SIM) method has been developed for simultaneous identification and quantification of **α**-asarone, *β*-asarone, and methyl eugenol of *Acorus tatarinowii* Schott in rat plasma. Chromatographic separation was performed on a Restek Rxi-5MS capillary column (30 m × 0.32 mm × 0.25 *μ*m), using 1-naphthol as internal standard (IS). MS detection of these compounds and IS was performed at *m/z* 178, 208, 208, and 144. Intra- and interday precisions of all compounds of interest were less than 10%. The recoveries are situated in the range of 92.4–105.2%. Pharmacokinetics of methyl eugenol confirmed to be one-compartment open model, **α**-asarone and **β**-asarone was two-compartment open model, respectively. The method will probably be an alternative to simultaneous determination and pharmacokinetic study of volatile ingredients in *Acorus tatarinowii* Schott.

## 1. Introduction

Chinese medicines have played an important role in clinical therapy in China, Korea, and Japan attributed to high pharmacological activity and few complications. As a classic medicinal plant, the dry rhizome of *Acorus tatarinowii* Schott is officially listed in the Chinese Pharmacopoeia and widely used for fighting ailments such as epilepsy, digestive disorders, cerebrovascular disease, stroke, senile dementia, and diarrhea [1–5]. Recent studies have found that aqueous extract and volatile oil of *Acorus tatarinowii* Schott dose-dependently suppressed the intensity of APO-induced stereotypic behavior and locomotor activity and extended the duration of sleeping induced by pentobarbital [6, 7]. Inhalation of the volatile oil also has the properties of reversing decreased brain GABA levels induced by PTZ inhibition of GABA transaminase [8]. 

Low polar or nonpolar compounds including *α*-asarone, *β*-asarone, and methyl eugenol proved to be main bioactive ingredients in volatile oil of *Acorus tatarinowii* Schott in previous report [9]. *β*-Asarone has significant pharmacological effects on cardiovascular and central nervous system, while *α*-asarone possesses hypocholesterolemic, hypolipidemic, neuroprotective, and antiepileptic activities [10–16]. Methyl eugenol was also found to have antinociceptive, antibacterial, and antidepressive activities [17, 18]. These compounds were often used as reference standards for quality control of *Acorus tatarinowii* Schott due to outstanding pharmacological activities. The development of sensitive and rapid approach for simultaneous determination of these compounds was owing to increasing interest in the literature. High performance liquid chromatography (HPLC), gas chromatography (GC), and GC with mass spectrometric detection (MS) were the main assays for analyzing *α*- and *β*-asarone in the medicinal plant and plasma in previous studies [19–23]. HPLC and MS method was also established for pharmacokinetics study of methyl eugenol in plasma [24, 25]. 

To our knowledge scope, few methods were found to be feasible for simultaneous determination of *α*-asarone, *β*-asarone, and methyl eugenol of *Acorus tatarinowii* Schott in biological samples, which produced issues of inaccurate evaluation of the medicinal plant and limitation of the pharmacokinetic study. This work was designed to develop a sensitive and valid method for determining *α*-asarone, *β*-asarone, and methyl eugenol of *Acorus tatarinowii* Schott in rat plasma. The work also aimed to apply the proposed assay in pharmacokinetic study of the three compounds after oral administration of volatile oil extracted from *Acorus tatarinowii* Schott.

## 2. Experimental

### 2.1. Chemicals and Reagents

*α*-Asarone (>98%) and 1-naphthol (>99%) were purchased from Sigma-Aldrich (St. Louis, MO, USA). Methyl eugenol (>99%) and *β*-asarone (>95%) were purchased from J&K Scientific Ltd (Guangdong, China). All other chemicals and reagents used in the experiment were of analytical grade unless stated specially. *Acorus tatarinowii* Schott was purchased from Xi'an Medical Company in China and identified by Professor Minfeng Fang in Northwest University.

The volatile oil of *Acorus tatarinowii* Schott was obtained by steam distillation of dry material of the plant. *Acorus tatarinowii* Schott (100.0 g) was put into 800 mL of distilled water and distilled for 8 h. The oil was collected from the condenser, dried by anhydrous sodium sulphate. The content of the oil in *Acorus tatarinowii* Schott was calculated to be 1.03% (v/w), with a determination of 0.50% methyl eugenol, 74.01% *β*-asarone, and 11.41% *α*-asarone by GC-MS analysis through area normalization method. 

### 2.2. Preparation of Standard Solutions

#### 2.2.1. Internal Standard

A stock solution of 1-naphthol (1.0 mg/mL) for IS was prepared in acetone and diluted to a concentration of 2.0 *μ*g/mL with acetone.

#### 2.2.2. Standard Solutions

Stock solution of *α*-asarone, *β*-asarone, and methyl eugenol was prepared by dissolving the appropriate amount of the standards in acetone. Mixed standard solution was prepared using the above solutions to give concentrations of 50.0 *μ*g/mL for *α*-asarone, 50.0 *μ*g/mL for *β*-asarone, and 50.0 *μ*g/mL for methyl eugenol. All the solutions were stored at 4°C in amber glass tubes. 

### 2.3. GC-MS/SIM Conditions

GC-MS analyses were performed with a Shimadzu GC-MS QP2010 (Kyoto, Japan). Chromatographic separation was achieved on a Restek Rxi-5MS capillary column (30 m × 0.32 mm I.D., 0.25 *μ*m film thickness, Shimadzu, Japan) using nitrogen as carrier gas at 2 mL/min in a constant flow rate mode. The GC oven temperature was initially increased from 60°C to 120°C at a rate of 5°C/min, then elevated at a rate of at 2°C/min up to 150°C, and then raised to 240°C at a rate of 10°C/min, giving a total run time of 36 min. The temperatures of injector, interface, and ion source were 250, 280, and 230°C, respectively. Detection was operated by selected ionmonitoring (SIM) mode (70 eV, electron impact mode).

In the SIM mode, the peaks of *α*-asarone, *β*-asarone, methyl eugenol, and IS in plasma were identified by matching the retention time and the abundant ions of *m/z* 178 for methyl eugenol, *m/z* 208 for *α*-asarone, *β*-asarone, and *m/z* 144 for IS. The injection volume was 1.0 *μ*L. Data were collected using the GC-MS solution (Kyoto, Japan). 

### 2.4. Animals and Plasma Collection

Six healthy male Sprague-Dawley rats, weighing 250–280 g, were supplied by the Guangdong Laboratory Animals Monitoring Institute (Guangdong, China). The rats were housed in a standard animal holding room with normal access to food and water and 12 h light cycle. The animals were fasted overnight prior to dosing, with water allowance ad libitum. All animal experiments were carried out according to the Guidelines for the Care and Use of Laboratory Animals and were approved by the Animal Experimentation Ethics Committee. 

Drug-free rat plasma samples were obtained from the postorbital venous plexus veins of anesthetized animals and collected into heparinized tubes. All the blood samples were centrifuged for 10 min at 8000 rpm to yield the supernatant stored at −20°C until analysis.

### 2.5. Sample Preparation

In a 1.5 mL Eppendorf tube, 100 *μ*L of plasma was spiked with 10 *μ*L of 2.0 *μ*g/mL IS solution. An aliquot of 40 *μ*L 10% (V : V) trichloracetic acid water solution and 600 *μ*L acetonitrile was used to remove the proteins in the plasma through vortexed for 2 min followed by the centrifugation of 10 min at 8000 rpm. The supernatant was collected in a clean tube for next use, while the residue was extracted by 300 *μ*L acetic ether for three times. The acetic ether layer and the above supernatant were collected together and evaporated to dryness under a stream of nitrogen. The residue was dissolved in 100 *μ*L of acetone for further GC-MS analysis.

### 2.6. Method Validation

#### 2.6.1. Calibration and Detection Limits

The linearity of *α*-asarone, *β*-asarone, and methyl eugenol was investigated by replicate analyses of calibration solutions with different concentration ranging from 5 to 5000 ng/mL and evaluated by linear regression. The limit of detection (LOD) was calculated by sequential diluting the analytes until the ratio of signal to noise equals 3 : 1. The limit of quantitation (LOQ) was considered as ten times of the signal to noise ratio.

#### 2.6.2. Precision, Accuracy, and Stability

Quality control (QC) samples to determine the accuracy and precision of the method were independently prepared by dissolving the standard in plasma at low, medium, and high concentrations of 0.025, 0.25 and 2.5 *μ*g/mL. The intraday precision and accuracy were determined within one day by analyzing the QC samples (*n* = 6). The interday precision and accuracy were determined on five separated days using the same QC samples. Precision was reported as the relative standard deviation (RSD). Accuracy was expressed as the relative error (RE).

The stability of analytes in the plasma was evaluated using the QC samples in triplicate. Test conditions included 4 freeze-thaw cycles and room temperature stability (0, 2, 4, 8, 12, and 24 h). The stabilities were assessed by analysis of the RSD of measured samples.

#### 2.6.3. Recoveries and Matrix Effect

The extraction recovery of the three drugs was determined by calculating the peak areas obtained from blank plasma samples spiked with analyte before extraction with those from blank plasma samples, to which analytes were added after extraction. According to the guidance of USFDA, recovery experiments should be performed at three concentrations (low, medium, and high). This procedure was accordingly repeated for five replicates at three concentrations of 0.025, 0.25, and 2.5 *μ*g/mL. In order to test the matrix effect on the ionization of the analyte, that is, the potential ion suppression or enhancement due to the matrix components, the drugs at three concentration levels were added to the extract of 0.1 mL of blank plasma, evaporated and reconstituted with 0.1 mL of acetone; the corresponding peak areas (A) were compared with those of the drugs standard solutions evaporated directly and reconstituted with the same solvent (B). The ratio (A/B × 100) % was used to evaluate the matrix effect. By the same method, the matrix effect of internal standard was also evaluated.

### 2.7. Pharmacokinetics Analysis

The GC-MS method was successfully applied in the pharmacokinetic studies of *α*-asarone, *β*-asarone, and methyl eugenol in rats. Volatile oil of *Acorus tatarinowii* Schott was administered orally to the rat in a single dose of 0.2 g/kg. Blood samples were collected in heparinized tube at 0, 5, 10, 20, 30, 60, 90, 120, 180, 300, 480, 720, 960, and 1200 min after dose. All the samples were extracted and analyzed under the proposed condition.

### 2.8. Data Analysis

The Microsoft Excel Program Drug and Statistics 2.0 (T.C.M. Shanghai, China) was employed to process and calculate the pharmacokinetic parameters. The parameters of the area under curve (AUC), the maximum plasma concentration (*C*_max_) and the corresponding time (*t*_max_), the half-life of absorption (*t*_1/2_), and so forth were used to decrypt the pharmacokinetic properties of *α*-asarone, *β*-asarone, and methyl eugenol. Statistical analysis of the biological data was performed by the Student's *t*-test. All results were expressed as arithmetic mean ± standard deviation (SD). 

## 3. Results and Discussion

### 3.1. Preparation of the Plasma Samples

In order to quantify drugs in a plasma sample, it is often necessary to disrupt the protein-drug binding for the production of the drugs-free form. Acetonitrile and trichloracetic acid were separately evaluated for extracting *α*-asarone, *β*-asarone, and methyl eugenol from plasma. When choosing the trichloracetic acid, the recoveries of *α*-asarone and *β*-asarone were less than 50%. When choosing the acetonitrile, the recoveries of *α*-asarone and *β*-asarone were near 80%. The recoveries of methyl eugenol and IS just have minor change. Interestingly, a mixture of acetonitrile and trichloracetic acid (10%) in ratios of 15 : 1 was found to be efficient to accomplish the extracting task. The recoveries of analytes were more than 90%. This solvent was accordingly used to prepare the plasma samples in the present work. 

### 3.2. Calibration and Detection Limits

The linear regression analysis was constructed by plotting the peak area ratio of the three analytes to IS against the concentrations of *α*-asarone, *β*-asarone, and methyl eugenol in plasma samples, respectively, shown in Table 1. The regression equation of the curves and the correlation coefficients (*r*) was calculated to be *y* = 6.326*x* − 0.020 and *r* = 0.999 for *α*-asarone, *y* = 6.469*x* − 0.027 and *r* = 0.997 for *β*-asarone, and *y* = 5.874*x* + 0.014 and *r* = 0.999 for methyl eugenol, using weighted least squares linear regression (the weighing factor was 1/*C*). For each drug, six calibration curves containing six concentrations (5, 25, 50, 250, 500, 2500, and 5000 ng/mL) were linear within the concentration range of 5–5000 ng/mL. The CV of the three drugs at each level varied from 2.0 to 12.3. And the relative bias of *α*-asarone, *β*-asarone, and methyl eugenol from the theoretical value varied from −3.0 to 4.6. As a result, the calibration curves of the three compounds exhibited good linearity within the experimental range. The LOD for *α*-asarone, *β*-asarone, and methyl eugenol was calculated to be 1.9, 1.8, and 1.5 ng/mL. The LOQ of the tested drugs was 4.7, 4.9, and 4.2 ng/mL.

### 3.3. Precision, Accuracy, and Stability

The data from QC samples were calculated to estimate the intraday and interday precision and accuracy of the method. The results are presented in Table 2. It was found that the intraday precision for low, mid, and high QC levels of *α*-asarone was 7.08%, 3.24%, and 4.85%, respectively, and that of the interday analysis was 4.80%, 4.42%, and 7.99%, with an accuracy (RE) within −4.74 to 4.04%. The precision for *β*-asarone of the QCs was determined as 4.84%, 6.34% plus 4.99% for intraday and 6.20%, 3.75%, and 6.01% for interday. The accuracy was found ranging from −6.03 to 2.93%. For methyl eugenol, the intraday precisions were 5.18%, 6.69%, and 4.11%, while the interday precisions were 8.10%, 7.68%, and 4.74%. The accuracy of methyl eugenol ranged between −7.62 and 5.15% for the three levels of QC samples. The precision and accuracy of the present method conform to the criteria for the analysis of biological samples according to the guidance of USFDA, where the precision (RSD) determined at each concentration level is required not to exceed 15%.

The stabilities were demonstrated by analysis of the RSD of measured samples. The results indicated that analytes were stable at 2, 4, 8, 12, and 24 h at room temperature and during multiple freeze-thaw cycles (≥95.6%). The three compounds were believed to have good stability in 24 h at room temperature and during three freeze-thaw cycles.

### 3.4. Recoveries and Matrix Effect

**Figure 1:**
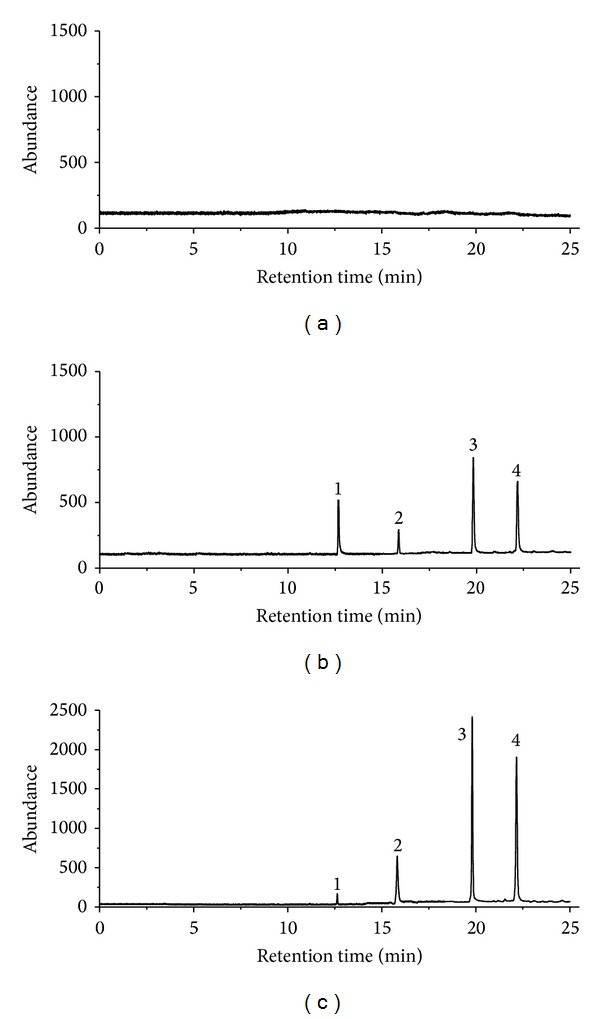
GC-MS/SIM chromatograms: (a) rat blank plasma sample; (b) standard solutions of methyl eugenol (0.2 *μ*g/mL), *β*-asarone (0.2 *μ*g/mL), *α*-asarone (0.2 *μ*g/mL), and IS (0.1 *μ*g/mL); (c) rat blood sample after a single oral administration of volatile oil of *Acorus tatarinowii* Schott. Peak 1: methyl eugenol; Peak 2: IS; Peak 3: *β*-asarone; Peak 4: *α*-asarone.

The results are presented in Table 3. The recoveries of *α*-asarone from rat plasma were 99.2 ± 7.0, 95.3 ± 3.1, and 100.7 ± 4.9% at concentration levels of 0.025, 0.25, and 2.5 *μ*g/mL, respectively, and the mean extraction recovery of IS was 93.6 ± 3.1%. The values for recoveries of *β*-asarone using the proposed method were 102.9 ± 5.0, 94.0 ± 6.0, and 95.1 ± 4.8%. For methyl eugenol, the extraction recoveries were calculated to be 105.2 ± 5.4, 92.4 ± 6.2, and 96.5 ± 4.0%. In terms of matrix effect, all the ratios (A/B × 100)% defined as in Section 2.6.3 were between 92.4 and 105.2%, which means no matrix effect for the three compounds and IS in using current method (Figure 1).

### 3.5. Pharmacokinetics Analysis

**Figure 2:**
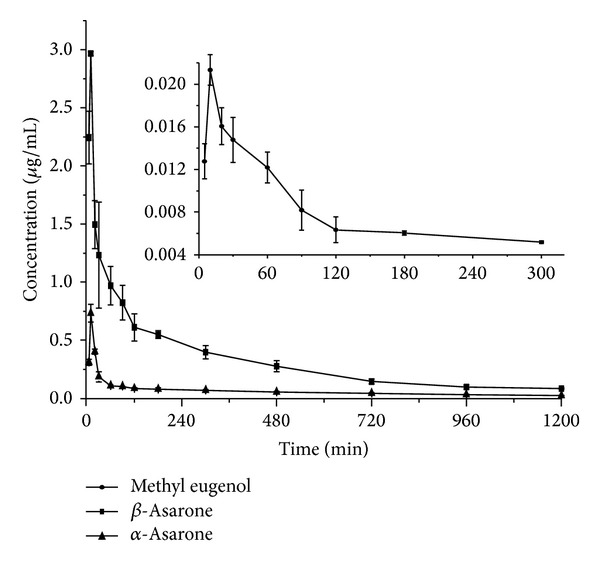
The mean plasma concentration-time profile of analytes in rats after oral administration volatile oil of *Acorus tatarinowii* Schott (0.2 g/kg).

The present method was applied to the pharmacokinetic investigations of *α*-asarone, *β*-asarone, and methyl eugenol following single oral dose of 0.2 g/kg volatile oil of *Acorus tatarinowii *Schott. Mean plasma drug concentration-time curve of the three compounds in single dose study was shown in Figure 2, and the calculating parameters were in Table 4. The results indicated that the plasma profile of methyl eugenol was confirmed to be one-compartment open models, while the pharmacokinetic behavior of *α*-asarone and *β*-asarone was in line with two-compartment open model. Nevertheless, after oral administration of the volatile oil, the *C*_max_ of the three drugs were calculated to be 0.021, 0.53, and 2.47 *μ*g/mL, with the *t*_max_ values of 10.00, 11.25, and 13.75 min. The AUC (0 ~ *∞*) were determined as 2.49, 56.67, and 407.52 mg/L· min, while the values of AUC (0 ~ *t*) were 2.13, 368.43, and 47.51 mg/L· min. The ratios of AUC $\begin{array}{c} \left(0\sim \infty \right) \end{array}$ versus AUC (0 ~ *t*) for the three drugs were all less than 120%, which indicated that the proposed blood collecting time was feasible to evaluate the pharmacokinetic behaviors of the three compounds.

## 4. Conclusions

A novel GC-MS/SIM method for simultaneously qualitative and quantitative analysis of *α*-asarone, *β*-asarone, and methyl eugenolin rat plasma is described in this work. The method combines the excellent power of separation performed by GC to the high sensitivity of MS detection system operating in SIM mode and it has the properties of high specificity, precision, accuracy, and stability. The method has been confirmed to be successful in pharmacokinetics study of *α*-asarone, *β*-asarone and methyl eugenol in *Acorus tatarinowii* Schott, which probably provides an alternative to monitor the three drugs in preclinical study.

## Figures and Tables

**Table 1:** Analytical parameters for GC-MS of plasma analytes assayed by the present method.

Analytes	Calibration curve^a^	Correlation coefficient	LOD (ng/mL)	LOQ (ng/mL)
Methyl eugenol	*y* = 5.874*x* + 0.014	0.999	1.553	4.236
*β*-Asarone	*y* = 6.469*x* − 0.027	0.997	1.802	4.872
*α*-Asarone	*y* = 6.326*x* − 0.020	0.999	1.685	4.719

^a^In the linear regression equation, *x* is expressed as analytes concentration (*μ*g/mL) and *y* is expressed as peak area of analytes/IS.

**Table 2:** Intraday (*n* = 6) and interday precision (*n* = 5) and accuracy of analytes from biological samples.

Analytes	Added (*μ*g/mL)	Precision (RSD, %)		Accuracy (RE, %)	
		Intraday	Interday	Intraday	Interday
Methyl eugenol	0.025	5.18	8.10	5.15	3.65
	0.250	6.69	7.68	−7.62	−2.15
	2.500	4.11	4.74	−3.54	−4.34

*β*-Asarone	0.025	4.84	6.20	2.89	2.93
	0.250	6.34	3.75	−6.03	−3.22
	2.500	4.99	6.01	−4.88	−5.76

*α*-Asarone	0.025	7.08	4.80	−0.77	−4.53
	0.250	3.24	4.42	−4.74	4.04
	2.500	4.85	7.99	0.71	−4.01

**Table 3:** Matrix effects and extraction recovery of analytes in biological samples.

Analyte	Concentration (*μ*g/mL)	Matrix effects (%, *n* = 6)	RSD (%)	Extraction recovery (%, *n* = 5)	RSD (%)
Methyl eugenol	0.025	93.8 ± 5.2	5.5	105.2 ± 5.4	5.1
	0.250	94.6 ± 5.8	6.1	92.4 ± 6.2	6.7
	2.500	105.2 ± 4.7	4.5	96.5 ± 4.0	4.1

*β*-Asarone	0.025	92.4 ± 6.6	6.5	102.9 ± 5.0	4.9
	0.250	98.6 ± 6.2	6.3	94.0 ± 6.0	6.4
	2.500	101.3 ± 3.8	3.8	95.1 ± 4.8	5.0

*α*-Asarone	0.025	96.9 ± 4.4	4.5	99.2 ± 7.0	7.1
	0.250	100.6 ± 7.8	7.8	95.3 ± 3.1	3.3
	2.500	103.8 ± 6.2	6.0	100.7 ± 4.9	4.9

I.S.	2.0	94.1 ± 4.0	4.3	93.6 ± 3.1	8.6

**Table 4:** Pharmacokinetic parameters of analytes in rats (*n* = 6).

Analytes	T_1/2 α_ (min)	*T*_1/2*β*_ (min)	*T*_1/2_ (min)	AUC_(0~*t*)_ (mg/L·min)	AUC_(0~*∞*)_ (mg/L·min)	CL (mL/min/kg)	Vd (L/kg)	*T*_max⁡_ (min)	*C*_max⁡_ (*μ*g/mL)
Methyl Eugenol			67.66 ± 3.32	2.13 ± 0.27	2.49 ± 0.25	0.40 ± 0.04	39.35 ± 4.08	10.00 ± 0.00	0.021 ± 0.001
*β*-Asarone	54.39 ± 29.46	69.30 ± 0.21		368.43 ± 40.05	407.52 ± 55.78	0.38 ± 0.05	149.87 ± 90.78	13.75 ± 11.09	2.47 ± 0.58
*α*-Asarone	58.92 ± 20.80	69.28 ± 6.12		47.51 ± 28.49	56.67 ± 35.91	0.60 ± 0.44	22.93 ± 16.11	11.25 ± 6.29	0.53 ± 0.24
